# Triglyceride-glucose index predicts postoperative delirium in elderly patients with type 2 diabetes mellitus: a retrospective cohort study

**DOI:** 10.1186/s12944-024-02084-2

**Published:** 2024-04-15

**Authors:** Miao Sun, Min Liu, Faqiang Zhang, Lijuan Sang, Yuxiang Song, Peng Li, Siyuan Liu, Huikai Yang, Libin Ma, Jiangbei Cao, Weidong Mi, Yulong Ma

**Affiliations:** 1 Department of Anesthesiology, The First Medical Center of Chinese PLA General Hospital, Beijing, 100730 China; 2 Nation Clinical Research Center for Geriatric Diseases, Chinese PLA General Hospital, Beijing, 100730 China; 3 Department of Anesthesiology, Beijing Tongren Hospital, Capital Medical University, Beijing, 100730 China; 4 Department of Anesthesiology, Shanghai Pulmonary Hospital, School of Medicine, Tongji University, Shanghai, 200433 China

**Keywords:** Insulin resistance, Triglyceride glucose index, Postoperative delirium, Type 2 diabetes mellitus, Elderly surgical patients

## Abstract

**Background:**

Postoperative delirium (POD) is more prevalent among elderly patients with type 2 diabetes mellitus (T2DM). Insulin resistance (IR) can be assessed using the triglyceride-glucose (TyG) index, a novel biomarker. This study aims to investigate the predictive potential of the TyG index for POD in elderly patients with T2DM.

**Materials and methods:**

Elderly patients (≥ 65) with T2DM who underwent non-neurosurgery and non-cardiac surgery were enrolled. Univariate and multivariate logistic regression analyses were conducted to assess the association between the TyG index and POD. Additionally, subgroup analyses were performed to compare the sex-specific differences in the predictive ability of the TyG index for POD.

**Results:**

A total of 4566 patients were included in this retrospective cohort. The receiver operating characteristic (ROC) curve analysis determined the optimal cut-off value for the TyG index to be 8.678. In the univariate model, a TyG index > 8.678 exhibited an odds ratio (OR) of 1.668 (95% CI: 1.210–2.324, *P* = 0.002) for predicting POD. In the multivariate regression models, the ORs were 1.590 (95% CI: 1.133–2.252, *P* < 0.008), 1.661 (95% CI: 1.199–2.325, *P* < 0.003), and 1.603 (95% CI: 1.137–2.283, *P* = 0.008) for different models. Subgroup analyses demonstrated that the predictive ability of the TyG index was more pronounced in females compared to males.

**Conclusion:**

The TyG index shows promise as a novel biomarker for predicting the occurrence of POD in elderly surgical patients with T2DM.

**Supplementary Information:**

The online version contains supplementary material available at 10.1186/s12944-024-02084-2.

## Introduction

Diabetes has emerged as a significant metabolic disease that poses a substantial threat to human life and health. As of 2021, the global prevalence of diabetes reached 536.6 million individuals, with diabetes-related healthcare expenditure exceeding $1 trillion [1]. It is estimated that approximately half of all diabetes patients will require surgery during their lifetime [2]. However, the long-term complications associated with diabetes, including inflammation, oxidative stress, vasculopathy, and renal insufficiency, significantly elevate the risk of postoperative complications, particularly in elderly patients [2, 3]. These complications have a considerable impact on patient prognosis and quality of life. Therefore, it is imperative to focus on prevention and treatment strategies for postoperative complications in elderly diabetic patients.

Postoperative delirium (POD), which is characterized by acute disturbances in attention and awareness, is a prevalent neurological complication among elderly surgical patients [4, 5]. POD has been linked to prolonged hospital stays, increased morbidity and mortality rates, and diminished quality of life [4, 5]. Prior studies have demonstrated a higher incidence of POD in elderly patients with diabetes compared with non-diabetics, which significantly affects their prognosis [6, 7]. However, limited research has investigated the risk factors associated with POD in this specific population. Identifying independent indicators for POD in elderly diabetic surgical patients is therefore crucial, as it could serve as a basis for developing novel perioperative interventions.

Type 2 diabetes mellitus (T2DM) accounts for 90–95% of all diabetes cases [4]. Insulin resistance (IR) underlies the pathogenesis of T2DM, reflecting reduced sensitivity of the body and tissues to insulin. Chronic IR can lead to central nervous system (CNS) dysfunction due to insulin’s critical role in neurosynaptic functioning, synaptic plasticity modulation, glucose uptake, and neuronal survival [8]. Previous research has established a connection between IR and Alzheimer’s disease and other neurodegenerative disorders [8]. However, the correlation between IR and POD in elderly patients with T2DM has not been extensively explored.

The “gold standard” for identifying IR is the hyperinsulinemic euglycemic clamp, but its application during the perioperative period is not practical [9]. The triglyceride-glucose (TyG) index, calculated by fasting glucose and triglyceride, has emerged as a promising surrogate marker of IR due to its strong correlation with the hyperinsulinemic euglycemic clamp [10]. Previous studies have shown that the TyG index can independently predict the incidence and prognosis of cardiovascular and cerebrovascular diseases [10–12]. However, the relationship between the TyG index and POD in elderly patients with T2DM has yet to be explored.

This study hypothesizes a correlation between TYG index and the incidence of POD in elderly patients with T2DM who undergo non-neurosurgery and non-cardiac surgery. The findings of this study indicated that elderly diabetic patients with higher TyG index were at greater risk of developing POD, and TyG index could be a novel predictor and intervention target for the prevention of POD in clinical practice.

## Materials and methods

### Study design and participants

The study protocol underwent review and approval by the institutional ethics committee of Chinese PLA General Hospital (No. S2019-311–03), and the informed consent was agreed to be waived by the institutional ethics committee of Chinese PLA General Hospital because the research did not involve any clinical treatment or individual privacy information. This retrospective cohort study adhered to the Strengthening the Reporting of Observational Studies in Epidemiology (STROBE) statement. Data were collected from the first medical center of the Chinese PLA General Hospital’s perioperative retrospective database, covering the period from January 2014 to April 2019. The inclusion criteria were as follows: (1) age ≥ 65 years; (2) patients with T2DM; (3) patients who underwent non-cardiac and non-neurosurgery procedures with anesthesia. The exclusion criteria were as follows: (1) patients who only underwent digestive endoscopy; (2) missing data on fasting blood glucose and fasting triglycerides before surgery; (3) missing data for over 50% of all variables.

### Outcome

The primary outcome of interest was the incidence of POD within 7 days following surgery. Cases of POD were recorded in the database of Anesthesiology department, as previously described [13]. POD was diagnosed by a neurologist based on descriptive words (mental status change, confusion, disorientation, agitation, delirium, inappropriate behaviour, inattention, hallucinations, and combative behaviour) and the postoperative drug regime (quetiapine, olanzapine, haloperidol, haloperidol, and risperidone) in the medical records according to the Diagnostic and Statistical Manual of Mental Disorders-IV criteria. The patients with presence of the above-mentioned symptoms and drugs in preoperative medical records were excluded.

### Data collection and definition of variables

Relevant data were extracted from the existing database. Potential confounding factors that could influence the incidence of POD were categorized as preoperative variables and intraoperative variables. The preoperative variables of interest included patient demographics: age, sex, body mass index (BMI), smoking, alcohol use, hypertension, cardiovascular diseases, chronic kidney diseases (CKD), chronic obstructive pulmonary diseases (COPD), cerebrovascular diseases, depression and anxiety), and the latest laboratory test results before surgery (fasting blood glucose, triglycerides, glycated serum protein (GSP), hemoglobin (Hb), white blood cell (WBC) count, platelets, albumin, serum creatinine (Cre), total cholesterol, low-density lipoprotein cholesterol (LDL), high-density lipoprotein cholesterol (HDL), alanine aminotransferase (ALT), aspartate aminotransferase (AST), total bilirubin, and prothrombin time (PT). The intraoperative variables included the type of surgery, type of anesthesia, duration of anesthesia, urine output, output, blood loss, administration of crystalloid and colloid solutions, dosage of dexmedetomidine, and duration of mean arterial pressure (MAP) < 60 mmHg. The TyG index was calculated by the formula:

$$\eqalign{& {\text{ln[fasting}}\,{\text{triglycerides}}\,({\text{mg}}/{\text{dL}}) \cr & \, \times \,{\text{fasting}}\,{\text{glucose}}\,({\text{mg}}/{\text{dL}})/2] \cr}$$

The completeness of the data is shown in Supplementary Tables 1, and all variables had a missing percentage of less than 20%. The missing continuous variables were imputed using the median imputation method, while the missing discrete variables were imputed using the mode imputation method.

### Statistical analysis

Data analysis was performed using Statistical Package for the Social Science (SPSS) version 26.0 (IBM, America) and R statistical software (R version 4.0.5, R Foundation for Statistical Computing, Australia).

Continuous variables with a normal distribution were presented as mean [standard deviation (SD)], while continuous variables with a skewed distribution were reported as median [interquartile range (IQR)]. Categorical data were summarized as counts and percentages. Student’s t-test was used to compare normally distributed continuous variables, while the Mann-Whitney U test was employed for intergroup comparisons of non-normally distributed continuous variables. The chi-squared test or Fisher’s exact test was used for inter-group comparisons of categorical variables. Moreover, the number of outcome events per variable (EPV) was taken into account, with a general minimum requirement of ten events [14]. According to the EPV method for determining sample size, our sample size was deemed sufficient for obtaining reliable estimates. In this study, the TyG index as a binary variable was used for the main focus of the research. Therefore, PASS version 15.0 was used to calculate the required sample size for a group allocation of 1:1 with α = 0.05, β = 0.20. And the POD incidences were 4.4% and 2.7% in higher TyG index group and lower TyG index group, respectively. Based on these parameters, the determined sample size of 1859 patients per group was identified.

Receiver operating characteristic (ROC) curve analysis was conducted to determine the optimal cut-off value of the TyG index for predicting POD. To explore the correlation between the TyG index and POD, univariate and multivariate logistic regression analyses were performed. The TyG index was examined as a continuous variable, binary variable based on ROC analysis, and multiple categories based on the interquartile range. In multivariate logistic regression analysis, variables with a *P*-value < 0.05 in univariate logistic regression were included. The inclusion of these significant covariates adheres to the principles of multicollinearity in the models. Collinearity was determined using variance inflation factor, and variables were accordingly removed from the final model (Variance Inflation Factor > 5). Model 1 adjusted for preoperative factors, including age, Hb, RBC count, WBC count, HDL, CKD, depression and anxiety. Model 2 adjusted for intraoperative factors, including emergency surgery, type of surgery, duration of anesthesia, urine output, blood loss, administration of crystalloid and colloid solutions, and duration of MAP < 60 mmHg. Model 3 adjusted for variables in Model 1 and Model 2. A two-sided *P*-value < 0.05 was considered statistically significant for all tests, except for the *P*-value < 0.10 in interaction analyse.

Restricted cubic splines (RCS) with five knots (5th, 27.5th, 50th, 72.5th, and 95th centiles) were performed to present the relationship between TyG index and the incidence of POD. The subgroup analysis of multivariate logistic regression in Model 3 was performed for female and male participants, considering potential sex differences in IR. To further explore the effect of gender on the TyG index to predict POD, the interaction analyse was also conducted.

The sensitivity analysis was conducted by E-value method on the website https://www.evalue-calculator.com/evalue/ with the data in Model 3 [15]. The E-value is used to quantify the degree to which an observed association between an exposure and an outcome could be explained by potential uncontrolled and unmeasured confounding.

Specifically, the E-value reflects the minimum strength of association that an unmeasured confounder would need to have with both the exposure and outcome, above and beyond the measured confounders, to fully explain away a specific estimated effect [16]. And reported ORs of identified risk factors for POD were compared with the E-value in present study [17–20]. Additional sensitivity analysis has also been conducted using the original, non-imputed data to detect if the results of TyG index as a binary variable are affected by imputing missing values.

## Results

### Participants characteristics of cohorts by TyG index

**Fig. 1:**
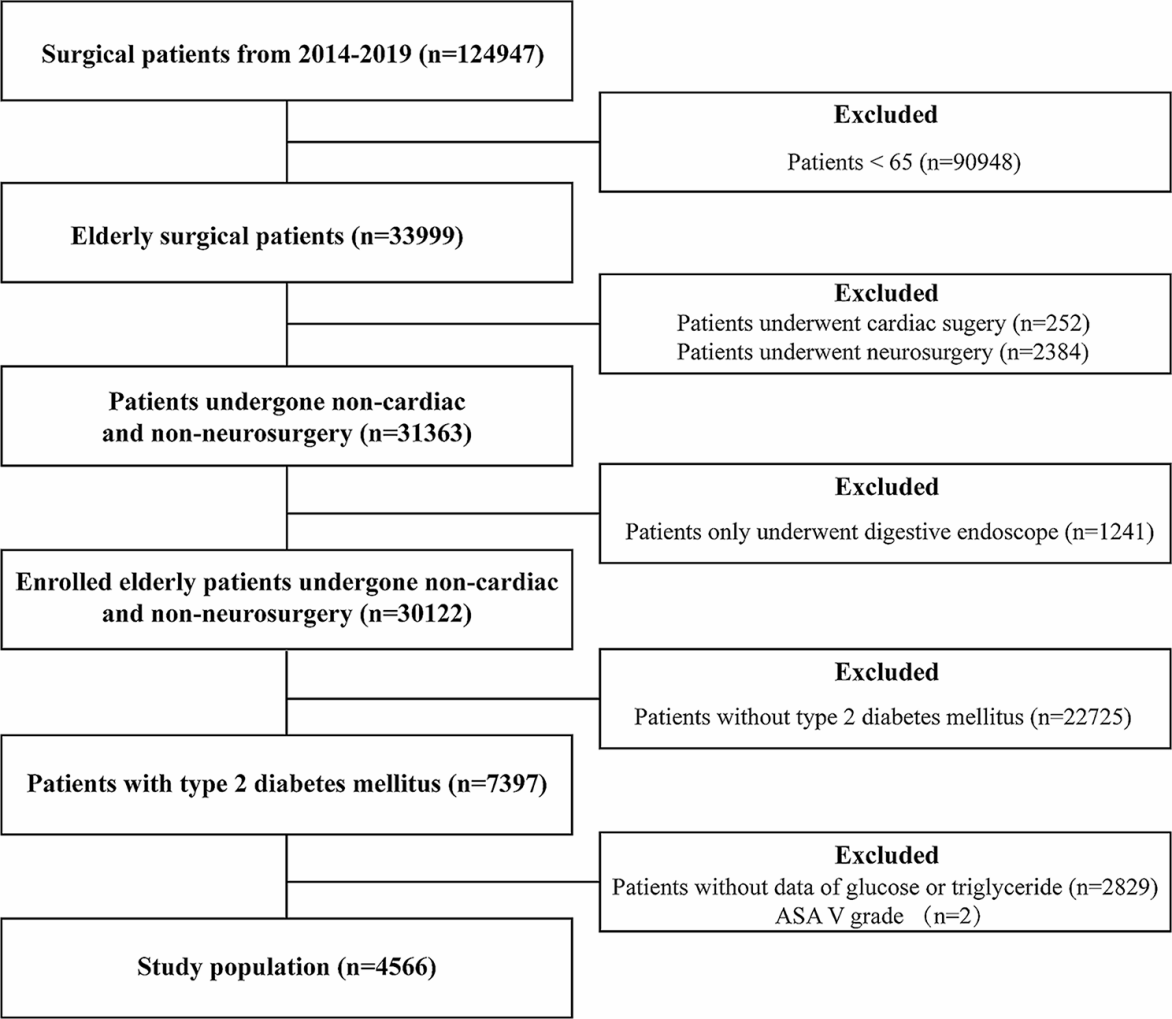
Flow chart of the study population. ASA, American society of anesthesiologists

**Fig. 2:**
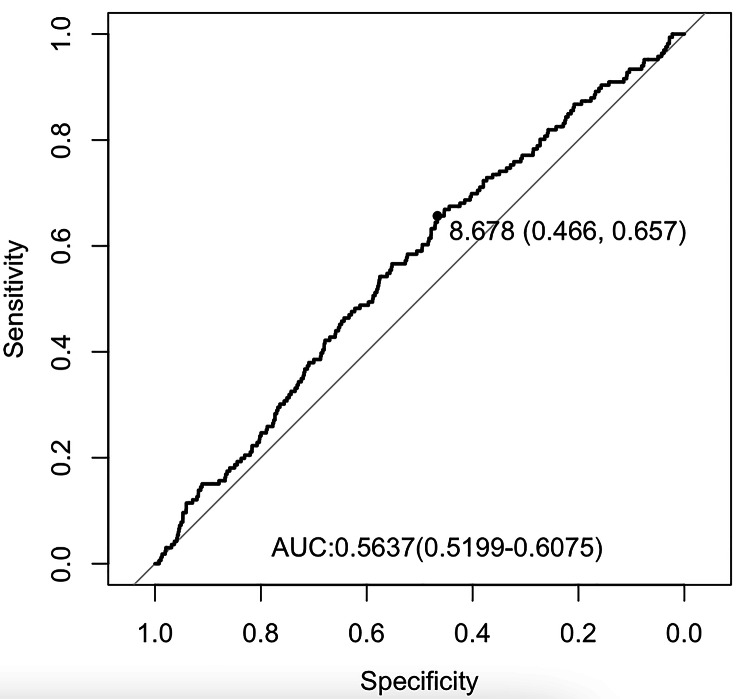
ROC curve of TyG index for predicting POD in surgical elderly patients with T2DM. The optimal cut-off point was 8.678 with specificity and sensitivity of 44.6% and 65.7% (area under the curve 0.5637, 95% CI: 0.5199 to 0.6075). ROC, receiver operating characteristic; TyG: triglyceride-glucose; POD, postoperative delirium; T2DM, type 2 diabetes mellitus; CI, confidence interval

A total of 4566 medical records of elderly patients were analyzed in this study (Fig. 1). The optimal cut-off value of the TyG index to predict POD was determined to be 8.678, with an area under the ROC curve (AUC) of 0.56 (Fig. 2). Table 1 presents the characteristics of the patients divided into two groups based on the critical value of the TyG index. The study included 2423 (53.1%) male participants and 2143 (46.93%) female participants. The median age of the participants was 70.0 years (interquartile range: 67.0–74.0), and the median TyG index was 8.74 (interquartile range: 8.34–9.17). The overall incidence of POD in the study participants was 3.6%. The group with TyG > 8.678 exhibited a higher incidence of POD, a higher proportion of female participants, lower prevalence of smoking and alcohol history, elevated levels of GSP and ALT, higher BMI, Hb, total cholesterol, low-density LDL, glucose, and triglyceride levels, lower levels of HDL, serum Cre, PT, and total bilirubin, and a younger age compared to the group with TyG < 8.678 (all *P* < 0.05). The clinical characteristics of participants by quartile of TyG index were presented in Supplementary Table 2.

**Table 1:** Clinical characteristics of participants by TyG index

Characteristics	Total	TyG ≤ 8.678	TyG > 8.678	*P* value
**Number, n (%)**	4566	2107	2459	
**POD, n (%)**				0.002
no	4400 (96.4)	2050 (97.3)	2350 (95.6)	
yes	166 (3.64)	57 (2.71)	109 (4.4)	
**Gender, n (%)**				< 0.001
**Male**	2423 (53.1)	1256 (59.6)	1167 (47.5)	
**Female**	2143 (46.9)	851 (40.4)	1292 (52.5)	
**Smoking, n (%)**				0.003
no	2423 (53.1)	1546 (73.4)	1900 (77.3)	
yes	2143 (46.9)	561 (26.6)	559 (22.7)	
**Alcohol, n (%)**				< 0.001
no	3446 (75.5)	1583 (75.1)	1983 (80.6)	
yes	1120 (24.5)	524 (24.9)	476 (19.4)	
**Hypertension, n (%)**				0.412
no	3566 (78.1)	664 (31.5)	804 (32.7)	
yes	1000 (21.9)	1443 (68.5)	1655 (67.3)	
**Cardiac disease, n (%)**				0.49
no	1468 (32.2)	599 (28.4)	723 (29.4)	
yes	3098 (67.8)	1508 (71.6)	1736 (70.6)	
**COPD, n (%)**				0.306
no	1322 (29.0)	2022 (96.0)	2375 (96.6)	
yes	3244 (71.0)	85 (4.0)	84 (3.4)	
**Cerebrovascular disease, n (%)**				0.531
no	4397 (96.3)	1830 (86.9)	2119 (86.2)	
yes	169 (3.7)	277 (13.1)	340 (13.8)	
**CKD, n (%)**				0.252
no	3949 (86.5)	2067 (98.1)	2399 (97.6)	
yes	617 (13.5)	40 (1.9)	60 (2.4)	
**Depression and anxiety, n (%)**				0.142
no	4466 (97.8)	2100 (99.7)	2442 (99.3)	
yes	100 (2.2)	7 (0.3)	17 (0.7)	
**ASA grade, n (%)**				0.054
I	40 (0.9)	17 (0.8)	23 (0.9)	
II	3329 (72.9)	1577 (74.8)	1752 (71.3)	
III	1166 (25.5)	501 (23.8)	665 (27.0)	
IV	31 (0.7)	12 (0.6)	19 (0.8)	
**Emergency surgery, n (%)**				0.056
no	4426 (96.9)	2054 (97.5)	2372 (96.5)	
yes	140 (3.1)	53 (2.5)	87 (3.5)	
**Surgical type, n (%)**				0.001
**Hepatopancreatobiliary and** **gastrointestinal surgery**	1455 (31.9)	691 (32.8)	764 (31.1)	
**Urinary surgery**	614 (13.4)	309 (14.7)	305 (12.4)	
**Thoracic surgery**	311 (6.8)	122 (5.8)	189 (7.7)	
**Gynecology**	186 (4.1)	84 (3.9)	102 (4.2)	
**E.N.T**	290 (6.4)	151 (7.2)	139 (5.7)	
**Vascular surgery**	229 (5.0)	114 (5.4)	115 (4.7)	
**Others**	1481 (32.4)	636 (30.2)	845 (34.4)	
**Anesthesia type, n (%)**				0.237
**General anesthesia**	4038 (88.4)	1876 (89.0)	2162 (87.9)	
**Basal anesthesia**	150 (3.3)	71 (3.4)	79 (3.2)	
**General anesthesia combined with other anesthesia**	288 (6.3)	120 (5.7)	168 (6.8)	
**Epidural anesthesia**	45 (1.0)	24 (1.1)	21 (0.9)	
**Nerve blocks**	45 (1.0)	16 (0.8)	29 (1.2)	
**GSP, µmol/L**	200 [178–226]	198 [170–212]	200 [189–237]	< 0.001
**Age, years**	70.0 [67.0–74.0]	71.0 [67.0–75.0]	70.0 [67.0–74.0]	< 0.001
**ALT, U/L**	14.9 [11.0-22.2]	14.1 [10.5–20.0]	15.8 [11.5–24.0]	< 0.001
**AST, U/L**	16.1 [13.3–20.6]	16.0 [13.4–20.0]	16.2 [13.2–21.2]	0.21
**BMI, kg/m** ^**2**^	24.8 [22.7–27.1]	24.2 [22.1–26.5]	25.3 [23.2–27.6]	< 0.001
**Hb, g/L**	130 [119–141]	129 [118–140]	130 [119–141]	0.002
WBC count, *10 ^**9**^ **/L**	6.04 [5.07–7.28]	5.81 [4.90–7.02]	6.23 [5.25–7.53]	< 0.001
**Total bilirubin, µmol/L**	10.5 [8.00–14.0]	10.8 [8.30–14.1]	10.2 [7.70–13.8]	0.002
**PT, s**	13.2 [12.7–13.8]	13.4 [12.9–13.9]	13.1 [12.6–13.6]	< 0.001
**Duration of anesthesia, min**	180 [115–245]	180 [115–241]	180 [119–250]	0.438
**Blood loss, ml**	80 [20–200]	50 [20–200]	100 [20–200]	0.524
**Urine, ml**	200 [50.0-500]	200 [50.0-500]	200 [50.0-500]	0.721
**Crystalloid, ml**	1350 [1000–2100]	1500 [1000–2100]	1300 [1000–2100]	0.606
**Colloid, ml**	500 [0-500]	500 [0-500]	500 [0-500]	0.828
**Cre, µmol/L**	71.2 [59.8–83.7]	72.1 [61.2–84.4]	70.4 [58.8–82.8]	< 0.001
**Total cholesterol, mmol/L**	4.22 [3.60–4.94]	4.00 [3.41–4.62]	4.44 [3.82–5.22]	< 0.001
**LDL, mmol/L**	2.64 [2.16–3.23]	2.56 [2.01–3.03]	2.70 [2.28–3.40]	< 0.001
**HDL, mmol/L**	1.10 [0.93–1.29]	1.16 [1.01–1.39]	1.06 [0.87–1.20]	< 0.001
**Glucose, mmol/L**	5.90 [4.96–7.34]	5.10 [4.53–5.87]	6.91 [5.76–8.49]	< 0.001
**Triglyceride, mmol/L**	1.27 [0.94–1.76]	0.93 [0.75–1.15]	1.69 [1.37–2.22]	< 0.001
**Duration of MAP < 60 mmHg, min**	5.0 [0.0–10.0]	5.0 [0.0–15.0]	5.0 [0.0–10.0]	0.06
Platelet count, *10 ^**9**^ **/L**	207 [170–252]	207 [168–251]	208 [171–253]	0.209

POD, postoperative delirium; COPD, chronic obstructive pulmonary disease; CKD, chronic kidney disease; ASA, American Society of Anesthesiologists; E.N.T., Otolaryngology head, and neck surgery; GSP, glycated serum protein; ALT, alanine aminotransferase; AST, aspartate aminotransferase; BMI, body mass index; Hb, hemoglobin; WBC, white blood cell; PT, prothrombin time; TyG, triglyceride-glucose; Cre, Creatinine; LDL, low density lipoprotein; HDL, high density lipoprotein; MAP, mean artery pressure

### Association between the TyG index and POD incidence

**Fig. 3:**
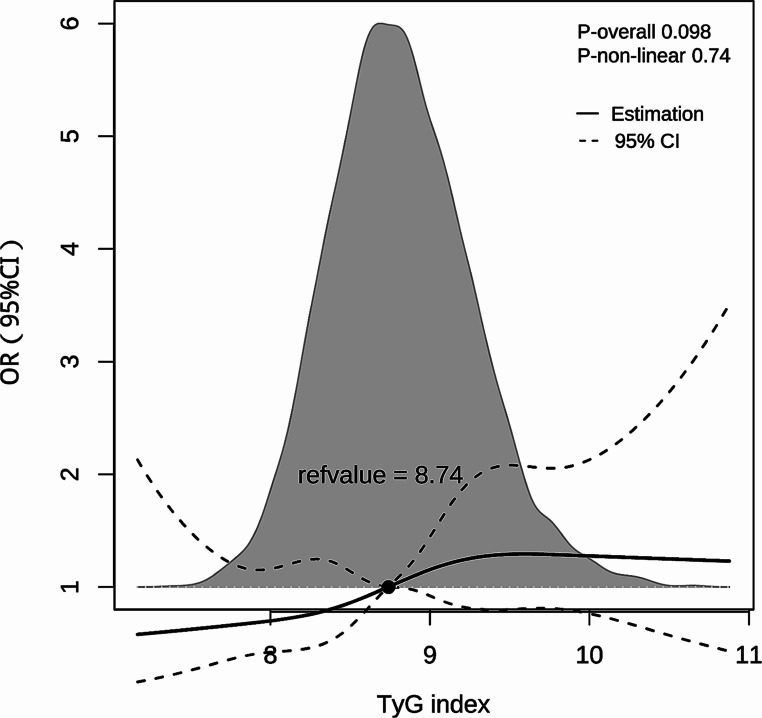
Restricted cubic spline plots. The relationship between the TyG index and OR of POD. OR, odd ratio; POD, postoperative delirium. The gray background shows the distribution of TyG index

The ROC curve analysis demonstrated the ability of the TyG index to predict the risk of POD (Fig. 2). And the RCS plots showed a “S-shape” association between TyG index and POD (Fig. 3). The *P* for non-linear relationship is 0.74, indicating the linear relationship between TyG index and POD incidence. The relationship between POD and the TyG index, grouped by the cut-off value, is presented in Table 2. In the univariate analysis (Supplementary Table 3), the odds ratio (OR) of the group with TyG > 8.678 was 1.668 (95% CI: 1.210–2.324, *P* = 0.002). In the multivariate logistic regression models (Supplementary Table 4), the adjusted ORs of the TyG > 8.678 group in model 1, model 2, and model 3 were 1.590 (95% CI: 1.133–2.252, *P* = 0.008), 1.661 (95% CI: 1.199–2.325, *P* = 0.003), and 1.603 (95% CI: 1.137–2.283, *P* = 0.008), respectively. These results demonstrate that a TyG > 8.678 is an independent risk factor for POD in elderly patients with T2DM.

**Table 2:** Association between TyG index and POD with logistic regression models

Model	OR^a^	95%CI	*P* value
**Unadjusted model**	1.668	1.210–2.324	0.002
**Model 1 (adjusted for preoperative variables)**	1.590	1.133–2.252	0.008
**Model 2 (adjusted for intraoperative variables)**	1.661	1.199–2.325	0.003
**Model 3 (adjusted for all the variables)**	1.603	1.137–2.283	0.008

OR^a^ is the odd ratio of TyG > 8.678; Preoperative variables include CKD, depression and anxiety, age, Hb, WBC, HDL, platelet count; Intraoperative variables include emergency surgery, surgery types, anesthesia time, blood loss, urine, Duration of MAP < 60 mmHg, Crystalloid, Colloid

The TyG index was also analyzed as a continuous variable. In the univariate analysis (Supplementary Table 3), the OR of the TyG index was 1.379 (95% CI: 1.084–1.749, *P* = 0.008). In the multivariate logistic regression models (Supplementary Table 5), the adjusted ORs of the TyG index in model 1, model 2, and model 3 were 1.268 (95% CI: 0.977–1.640, *P* = 0.072), 1.335 (95% CI: 1.047–1.698, *P* = 0.019), and 1.255 (95% CI: 0.962–1.631, *P* = 0.092), respectively. Notably, as a continuous variable, the TyG index was only significant in model 2, which included intraoperative variables.

The participants were further divided into four groups (group 1: TyG ≤ 8.338, group 2: 8.338 < TyG ≤ 8.736, group 3: 8.736 < TyG ≤ 9.171, group 4: TyG > 9.171) based on the quartiles of the TyG index levels (Supplementary Table 6). Group 1 served as the reference. In the univariate analysis (Supplementary Table 3), the incidence of POD increased with higher TyG index levels, and the OR of group 4 was 1.728 (95% CI: 1.100-2.762, *P* = 0.019). In the multivariate logistic regression models, the adjusted ORs of group 4 in model 1, model 2, and model 3 were 1.518 (95% CI: 0.936–2.498, *P* = 0.095), 1.666 (95% CI: 1.055–2.677, *P* = 0.031), and 1.489 (95% CI: 0.913–2.464, *P* = 0.115), respectively. Similar to the results obtained using the using the TyG index as a continuous variable to predict POD, only group 4 of the TyG index in model 2 showed significance in the multivariate logistic regression analysis.

### Subgroup analysis for sex

The comparison between male and female participants with respect POD incidence, age, comorbidities (cerebrovascular disease, chronic kidney disease, depression, and anxiety), American Society of Anesthesiologists (ASA) grade, AST, urine output, albumin level, glucose level, emergency surgery, blood loss, duration of MAP < 60 mmHg, and platelet count revealed no significant differences (all *P* > 0.05) (Supplementary Table 7). However, compared to males, females had a higher prevalence of hypertension and cardiac disease, elevated BMI, TyG index, total cholesterol, LDL, HDL, and triglyceride levels, lower prevalence of COPD, GSP, ALT, Hb, WBC count, total bilirubin, and Cre levels, shorter PT and duration of anesthesia, and received lesser volumes of crystalloid and colloid solutions (all *P* < 0.05).

To explore the differences between males and females in the relationship between the TyG index and POD incidence, a subgroup analysis was conducted (Table 3). In male patients, the relationship between the TyG index and POD incidence was not significant in any of the models. In contrast, the adjusted OR of the TyG index as a continuous variable in females was 1.95 (95% CI: 1.28–2.96, *P* < 0.001), and the adjusted OR of TyG index > 8.678 in females was 2.24 (95% CI: 1.25–4.19, *P* = 0.01). When analyzed based on interquartile groups of the TyG index, the adjusted OR of group 4 in females was 2.68 (95% CI: 1.18–6.78, *P* = 0.03). The *P* values for interaction analyses between gender and TyG index as a continuous variable, a binary variable and a quartile variable in Model 3 were 0.067, 0.435 and 0.046, respectively. There is interaction effect between gender and TyG index to predict POD when TyG acts as a continuous and a quartile bariable. These results from the subgroup analysis indicate that the TyG index is a more effective predictor of POD incidence in females among elderly patients with T2DM.

**Table 3:** Subgroup analysis for sex

Subgroup	POD incidence (%)	OR (95CI%)	*P* value
Female			
**TyG as continuous variable**	3.9	1.95 (1.28–2.96)	< 0.001
**TyG ≤ 8.678**	2.1	Reference	
**TyG > 8.678**	4.1	2.24 (1.25–4.19)	0.01
**TyG ≤ 8.338**	1.9	Reference	
**8.338 < TyG ≤ 8.736**	2.6	1.18 (0.47–3.16)	0.72
**8.736 < TyG ≤ 9.171**	3.2	1.78 (0.76–4.55)	0.20
**TyG > 9.171**	5.0	2.68 (1.18–6.78)	0.03
**Male**			
**TyG as continuous variable**	3.3	1.00 (0.69–1.44)	0.99
**TyG ≤ 8.678**	3.1	Reference	
**TyG > 8.678**	4.8	1.47 (0.93–2.34)	0.10
**TyG ≤ 8.338**	3.1	Reference	
**8.338 < TyG ≤ 8.736**	3.9	1.30 (0.71–2.40)	0.40
**8.736 < TyG ≤ 9.171**	5.1	1.68 (0.91–3.12)	0.09
**TyG > 9.171**	3.9	1.03 (0.51–2.06)	0.93

TyG, triglyceride-glucose; POD, postoperative delirium

### Sensitivity analysis

The E-value is established as 2.59 in present study due to the OR of TyG index > 8.678 to predict POD in Model 3. As ORs of most risk factors for POD were less than 2.59 in other reported studies, the results of present study could be considered to have good robustness.

As shown in Supplementary Table 8, OR of the group with TyG > 8.678 in the univariate analysis was 1.593 (95% CI: 1.146–2.238, *P* = 0.006). In the multivariate logistic regression models, the adjusted ORs of the TyG > 8.678 group in model 1, model 2, and model 3 were 1.493 (95% CI: 1.054–2.133, *P* = 0.025), 1.648 (95% CI: 1.763–2.331, *P* = 0.004), and 1.582 (95% CI: 1.108–2.279, *P* = 0.012), respectively. These sensitivity analysis has strengthened the the validity of results that TyG index has a effective prediction for POD in elderly patients with T2DM.

## Discussion

POD is a common neurological complication among elderly surgical patients, and evidence suggests that patients with T2DM have a 1.6 times higher risk of developing POD compared to non-diabetic patients [4, 21]. Therefore, it is crucial to identify targetable risk factors or indices to reduce the incidence of POD and improve outcomes in elderly patients with T2DM. In the current study, the TyG index was found to be an independent risk factor for POD in this patient population.

IR plays a significant role in promoting surgery-induced systemic inflammation, which is a primary cause of POD [22]. Pathological mechanisms linking IR to cognitive impairment include dysregulation of the insulin signaling pathway affecting hippocampal plasticity, amyloid precursor protein metabolism, tau protein deposition, neuroinflammation, and apolipoprotein E ε4 allele expression [23]. Additionally, insulin plays a role in controlling cerebral vascular function, and reduced brain vasoreactivity during IR can lead to vascular cognitive impairment [23]. Clinical evidence supports the association between higher IR levels in elderly patients and an increased risk of postoperative cognitive impairment [9]. Conversely, interventions targeting IR, such as intranasal insulin treatment and exercise, have demonstrated cognitive function improvements in clinical and animal experiments [24–27]. Therefore, IR represents a potential pathological mechanism underlying POD in elderly patients with T2DM [28].

The TyG index is a novel biomarker that positively correlates with the degree of IR [29]. Its mechanism of indicating IR involves enhanced fat mobilization and lipid overload in the circulation [29]. Increased levels of free fatty acids and their metabolites accumulate in the liver, muscle, and pancreatic beta cells, inhibiting the insulin pathway [30, 31]. Moreover, the production of glycerol 3-phosphate leads to elevated blood sugar levels, and prolonged pathological states can result in IR [32]. While other methods exist to detect IR, such as the frequently sampled intravenous glucose tolerance test, oral glucose tolerance test, mixed meal tolerance test, insulin suppression test, and homeostatic model assessment (HOMA), the TyG index is more suitable for clinical application [33]. Previous measurements often require additional blood collection to determine insulin or C-peptide levels, which are not part of routine preoperative biochemical examinations. In contrast, the TyG index can be conveniently calculated using fasting plasma glucose and triglyceride levels, which are commonly tested preoperatively. Therefore, the TyG index serves as an ideal clinical marker for assessing perioperative IR in surgical patients.

Previous studies have shown that the TyG index can predict major adverse cardiovascular events in patients with T2DM [34, 35]. It has also been associated with an increased risk of long-term dementia and cognitive impairment following cerebral vessel disease in T2DM patients [36]. Furthermore, the TyG index has demonstrated better predictive performance than traditional indices such as HOMA-IR in neural disorders [37]. However, the relationship between the TyG index and POD in T2DM patients remains unclear. The present study is the first to reveal a significant association between a TyG index > 8.678 and increased incidence of POD in elderly patients with T2DM, suggesting it could serve as a new biochemical threshold for preoperative interventions in this population. According to the RCS curve, the relationship between TyG index and POD showed a “S-shape”. In the range of TyG index values below 9, the incidence of postoperative delirium (POD) increases as the TyG index increases. However, in the range of TyG index values above 9, there is no significant change in the incidence of POD. What’s more, the interaction effect of gender on TyG index was significant when TyG index was analyzed as a continuous variable and a quartile variable. This may be the reason for the better predictive ability of the TyG index for POD when acts as a binary variable.

Surgical patients with T2DM are prone to hyperglycemia due to IR, and intensive glucose control during the perioperative period has been shown to reduce the incidence of POD in diabetic patients [38]. However, strict glucose control to lower glucose levels may increase the risk of hypoglycemia, and the optimal standard for perioperative glucose levels remains unknown. Consequently, managing glucose levels alone is insufficient to address the risk of POD in patients with diabetes, and it is proposed that the TyG index should be screened in clinical practice to assess the risk of POD in elderly patients with T2DM.

Interestingly, this study found that the TyG index demonstrated better predictive performance for POD in females compared to males. This finding may be attributed to the fact that females are more likely to develop insulin resistance after the age of fifty [39], and estrogens have a potential protective effect on the body’s insulin sensitivity [40]. Consistent with current study, females had been observed to have significantly higher average TyG index and BMI values, as well as markedly higher incidences of hypertension and cardiac disease compared to males. These findings suggest that estrogen deficiency may promote insulin resistance in elderly women. However, there was no statistically significant difference in the incidence of POD between males and females. This may be due to the presence of different risk factors for POD between the sexes. Further research is warranted to explore potential differences in cognitive function between males and females. Some studies have reported a significant association between high TyG index in females and subclinical atherosclerosis [41]. In the subgroup analysis stratified by sex, a significant relationship was found between the TyG index and POD in females across all three models, which included TyG index as two categories, four categories, and continuous variables. This highlights the influence of gender on the relationship between insulin resistance and the prediction of POD.

### Study strengths and limitations

Firstly, the study included 4566 elderly patients aged 65 or above with T2DM, which is more targeted on high-risk individuals for POD. Secondly, the study used univariate and multivariate logistic regression analyses to thoroughly evaluate the association between the TyG index and POD. Thirdly, composed of clinical routine indicators, the TyG index is easy to implement and promote for the prevention of POD.

Despite the valuable insights gained from present study, there are several limitations that should be acknowledged. Firstly, the current study was retrospective in nature, and the assessment of POD relied on medical and nursing records instead of standardized scales like the 3-minute Diagnostic Interview for Confusion Assessment Method (3D-CAM). This might have led to an measurement bias of POD incidence compared to the actual situation. Further more, it is possible that unaccounted confounding factors may have influenced the observed association between TyG and POD in our study and the cross-sectional design of the study suggests that the associations observed in the data do not necessarily imply causality. Secondly, given the AUC value, it appears that the effectiveness of the TyG index as a predictive tool might still be constrained. Due to the multitude of etiologies and risk factors for delirium, as well as its varied clinical presentations, it is difficult to explain the occurrence and progression of delirium with a single pathological mechanism. What’s more, our study population consists of patients with T2DM, who are already at a high risk for POD and are susceptible to multiple high-risk factors for POD. Thirdly, the impact of the duration of diabetes onset and the use of diabetes medications had not been analyzed, as extracting relevant data from medical records was proved to be challenging.

## Conclusion

The TyG index holds promise as a potential biomarker for predicting POD in elderly surgical patients with T2DM, with a stronger predictive performance observed in females compared to males. Furthermore, a comprehensive consideration of blood glucose and triglyceride levels provides a new perspective and strategy for anesthesiologists in the preoperative management and prevention of POD in elderly patients with T2DM.

### Electronic supplementary material

Below is the link to the electronic supplementary material.

Supplementary Material 1

Supplementary Material 2

Supplementary Material 3

Supplementary Material 4

Supplementary Material 5

Supplementary Material 6

Supplementary Material 7

Supplementary Material 8

## Data Availability

No datasets were generated or analysed during the current study.

## Abbreviations

POD: Postoperative delirium
T2DM: type 2 diabetes mellitus
IR: Insulin resistance
TyG: Triglyceride-glucose
ROC: Receiver operating characteristic
OR: Odds ratio
CNS: Central nervous system
COPD: Chronic obstructive pulmonary disease
CKD: Chronic kidney disease
ASA: American Society of Anesthesiologists
E.N.T.: Otolaryngology head, and neck surgery
GSP: Glycated **s**erum protein
ALT: Alanine aminotransferase
AST: Aspartate aminotransferase
BMI: Body mass index
Hb: Hemoglobin
WBC: White blood cell
PT: Prothrombin time
Cre: Creatinine
LDL: Low density lipoprotein
HDL: High density lipoprotein
MAP: Mean artery pressure
SPSS: Statistical Package for the Social Science
SD: standard deviation
IQR: interquartile range
HOMA: homeostatic model assessment
3D-CAM: 3-minute Diagnostic Interview for Confusion Assessment Method
RCS: restricted cubic spline
