# Correction: Triglyceride-glucose index predicts postoperative delirium in elderly patients with type 2 diabetes mellitus: a retrospective cohort study

**DOI:** 10.1186/s12944-024-02120-1

**Published:** 2024-04-30

**Authors:** Miao Sun, Min Liu, Faqiang Zhang, Lijuan Sang, Yuxiang Song, Peng Li, Siyuan Liu, Huikai Yang, Libin Ma, Jiangbei Cao, Weidong Mi, Yulong Ma

**Affiliations:** 1 Department of Anesthesiology, The First Medical Center of Chinese PLA General Hospital, Beijing, 100730 China; 2 Nation Clinical Research Center for Geriatric Diseases, Chinese PLA General Hospital, Beijing, 100730 China; 3 Department of Anesthesiology, Beijing Tongren Hospital, Capital Medical University, Beijing, 100730 China; 4 Department of Anesthesiology, Shanghai Pulmonary Hospital, School of Medicine, Tongji University, Shanghai, 200433 China

**Correction** **:** **Lipids Health Dis 23, 107 (2024)**

**https://doi.org/10.1186/s12944-024-02084-2**

Following publication of the original article [1], the authors noticed that Figure 2 is incorrect. The correct figure 2 is shown below.

**Fig. 2:**
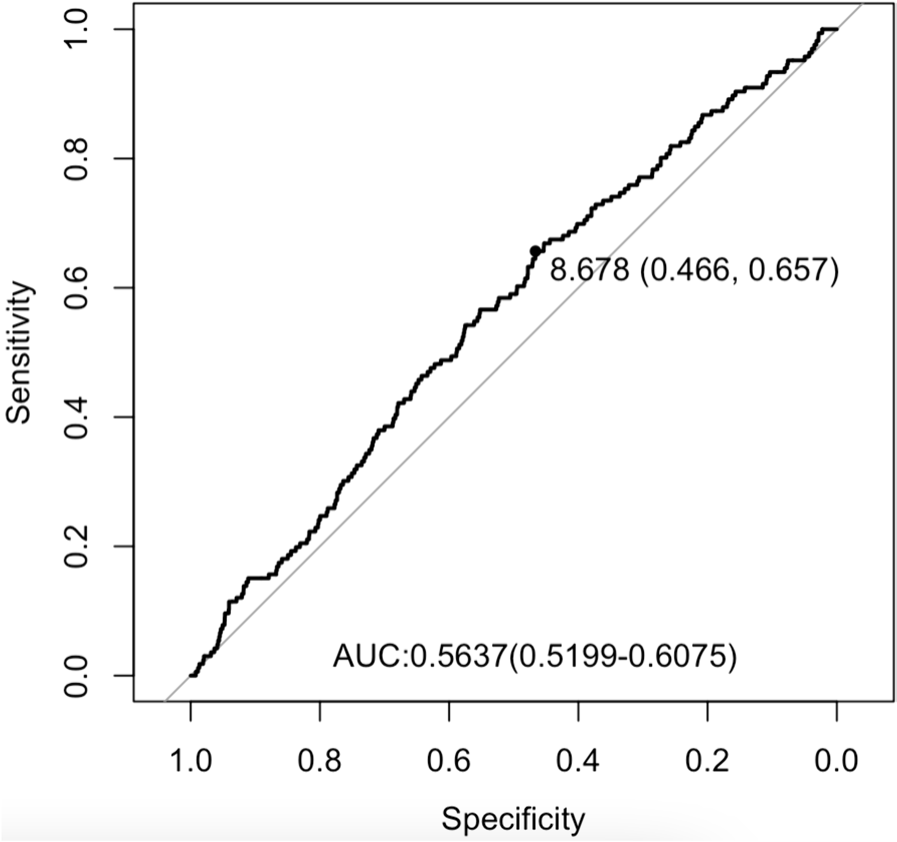
ROC curve of TyG index for predicting POD in surgical elderly patients with T2DM. The optimal cut-off point was 8.678 with specificity and sensitivity of 44.6% and 65.7% (area under the curve 0.5637, 95% CI: 0.5199 to 0.6075). ROC, receiver operating characteristic; TyG: triglyceride-glucose; POD, postoperative delirium; T2DM, type 2 diabetes mellitus; CI, confidence interval

The original article [1] has been updated.
