# Supplementary material for: Triglyceride-glucose index predicts postoperative delirium in elderly patients with type 2 diabetes mellitus: a retrospective cohort study
Source: Lipids Health Dis. 2024 Apr 15;23:107. doi: 10.1186/s12944-024-02084-2 (PMC11017528; doi:10.1186/s12944-024-02084-2)
Supplement: Supplementary file 1 — Supplementary Material 1 [file 12944_2024_2084_MOESM1_ESM.doc]

**Supplementary table 1. Number and percentage of missing data for each variable**

| **Characteristics** | **Number，n(%)** |
| --- | --- |
| **POD** | 0 (0.0) |
| **Smoking** | 0 (0.0) |
| **Alcohol** | 0 (0.0) |
| **Hypertension** | 0 (0.0) |
| **Cardiac disease** | 0 (0.0) |
| **COPD** | 0 (0.0) |
| **Cerebrovascular disease** | 0 (0.0) |
| **CKD** | 0 (0.0) |
| **Depression and anxiety** | 0 (0.0) |
| **ASA grade** | 0 (0.0) |
| **Emergency surgery** | 0 (0.0) |
| **Surgical type** | 0 (0.0) |
| **Anesthesia type** | 0 (0.0) |
| **GSP** | 813 (17.8) |
| **Age** | 0 (0.0) |
| **ALT** | 1 (<0.1) |
| **AST** | 4 (<0.1) |
| **BMI** | 7 (0.1) |
| **Hb** | 3 (<0.1) |
| **WBC count** | 4 (<0.1) |
| **Total bilirubin** | 13 (0.3) |
| **PT** | 7 (0.2) |
| **Duration of anesthesia** | 65 (1.4) |
| **Blood loss** | 22 (0.5) |
| **Urine** | 16 (0.4) |
| **Crystalloid** | 28 (0.6) |
| **Colloid** | 23 (0.5) |
| **Cre** | 0 (0.0) |
| **Total cholesterol** | 6 (0.13) |
| **LDL** | 288 (6.31) |
| **HDL** | 302 (6.6) |
| **Glucose** | 0 (0.0) |
| **Triglyceride** | 0 (0.0) |
| **Duration of MAP<60.0 mmHg** | 0 (0.0) |
| **Platelet count** | 25 (0.6) |

POD, postoperative delirium; COPD, chronic obstructive pulmonary disease; CKD, chronic kidney disease; ASA, [American Society of Anesthesiologists;](https://www.medsci.cn/guideline/search?keyword=美国麻醉医师协会(ASA,American Society of Anesthesiologists)) E.N.T., Otolaryngology head, and neck surgery; GSP, glycated serum protein; ALT, alanine aminotransferase; AST, aspartate aminotransferase; BMI, body mass index; Hb, hemoglobin; WBC, white blood cell; PT, prothrombin time; TyG, triglyceride-glucose; Cre,Creatinine; LDL, low density lipoprotein; HDL, high density lipoprotein; MAP, mean artery pressure.
