# Supplementary material for: Triglyceride-glucose index predicts postoperative delirium in elderly patients with type 2 diabetes mellitus: a retrospective cohort study
Source: Lipids Health Dis. 2024 Apr 15;23:107. doi: 10.1186/s12944-024-02084-2 (PMC11017528; doi:10.1186/s12944-024-02084-2)
Supplement: Supplementary file 2 — Supplementary Material 2 [file 12944_2024_2084_MOESM2_ESM.doc]

**Supplementary Table 2. Clinical characteristics of participants by quartile of TyG index**

| **Characteristics** | **Total** | **TyG ≤ 8.338** | **8.338 < TyG ≤ 8.736** | **8.736 < TyG ≤ 9.171** | **TyG > 9.171** | ***P* value** |
| --- | --- | --- | --- | --- | --- | --- |
| **Number, n (%)** | 4566 | 1139 (25.0) | 1137 (24.9) | 1148 (25.1) | 1142 (25.0) |  |
| **POD, n (%)** |  |  |  |  |  | 0.091 |
| no | 4400 (96.4) | 1109 (97.4) | 1099 (96.7) | 1101 (95.9) | 1091 (95.5) |  |
| yes | 166 (3.64) | 30 (2.6) | 38 (3.3) | 47 (4.1) | 51 (4.5) |  |
| **Gender, n (%)** |  |  |  |  |  | <0.001 |
| **Male** | 2423 (53.1) | 713 (62.6) | 640 (56.3) | 552 (48.1) | 518 (45.4) |  |
| **Female** | 2143 (46.9) | 426 (37.4) | 497 (43.7) | 596 (51.9) | 624 (54.6) |  |
| **Smoking, n (%)** |  |  |  |  |  | 0.009 |
| no | 2423 (53.1) | 822 (72.2) | 854 (75.1) | 879 (76.6) | 891 (78.0) |  |
| yes | 2143 (46.9) | 317 (27.8) | 283 (24.9) | 269 (23.4) | 251 (22.0) |  |
| **Alcohol, n (%)** |  |  |  |  |  | <0.001 |
| no | 3446 (75.5) | 830 (72.9) | 887 (78.0) | 912 (79.4) | 937 (82.0) |  |
| yes | 1120 (24.5) | 309 (27.1) | 250 (22.0) | 236 (20.6) | 205 (18.0) |  |
| **Hypertension, n (%)** |  |  |  |  |  | 0.928 |
| no | 3566 (78.1) | 359 (31.5) | 365 (32.1) | 369 (32.1) | 375 (32.8) |  |
| yes | 1000 (21.9) | 780 (68.5) | 772 (67.9) | 779 (67.9) | 767 (67.2) |  |
| **Cardiac disease, n (%)** |  |  |  |  |  | 0.977 |
| no | 1468 (32.2) | 324 (28.4) | 330 (29.0) | 334 (29.1) | 334 (29.2) |  |
| yes | 3098 (67.8) | 815 (71.6) | 807 (71.0) | 814 (70.9) | 808 (70.8) |  |
| **COPD, n (%)** |  |  |  |  |  | 0.094 |
| no | 1322 (29.0) | 1101 (96.7) | 1084 (95.3) | 1116 (97.2) | 1096 (96.0) |  |
| yes | 3244 (71.0) | 38 (3.3) | 53 (4.7) | 32 (2.8) | 46 (4.0) |  |
| **Cerebrovascular disease, n (%)** |  |  |  |  |  | 0.260 |
| no | 4397 (96.3) | 1000 (87.8) | 978 (86.0) | 999 (87.0) | 972 (85.1) |  |
| yes | 169 (3.7) | 139 (12.2) | 159 (14.0) | 149 (13.0) | 170 (14.9) |  |
| **CKD, n (%)** |  |  |  |  |  | 0.101 |
| no | 3949 (86.5) | 1120 (98.3) | 1112 (97.8) | 1127 (98.2) | 1107 (96.9) |  |
| yes | 617 (13.5) | 19 (1.7) | 25 (2.2) | 21 (1.8) | 35 (3.1) |  |
| **Depression and anxiety, n (%)** |  |  |  |  |  | 0.508 |
| no | 4466 (97.8) | 1134 (99.6) | 1133 (99.6) | 1142 (99.5) | 1133 (99.2) |  |
| yes | 100 (2.2) | 5 (0.4) | 4 (0.4) | 6 (0.5) | 9 (0.8) |  |
| **ASA grade, n (%)** |  |  |  |  |  | 0.093 |
| Ⅰ | 40 (0.9) | 10 (0.9) | 9 (0.79) | 9 (0.8) | 12 (1.1) |  |
| Ⅱ | 3329 (72.9) | 855 (75.1) | 845 (74.3) | 840 (73.2) | 789 (69.1) |  |
| Ⅲ | 1166 (25.5) | 270 (23.7) | 274 (24.1) | 289 (25.2) | 333 (29.1) |  |
| Ⅳ | 31 (0.7) | 4 (0.3) | 9 (0.8) | 10 (0.8) | 8 (0.7) |  |
| **Emergency surgery, n (%)** |  |  |  |  |  | 0.426 |
| no | 4426 (96.9) | 1106 (97.1) | 1109 (97.5) | 1110 (96.7) | 1101 (96.4) |  |
| yes | 140 (3.1) | 33 (2.9) | 28 (2.5) | 38 (3.3) | 41 (3.6) |  |
| **Surgical type, n (%)** |  |  |  |  |  | 0.043 |
| **Hepatopancreatobiliary and**  **gastrointestinal surgery** | 1455 (31.9) | 375 (32.9) | 365 (32.1) | 361 (31.4) | 354 (31.0) |  |
| **Urinary surgery** | 614 (13.4) | 166 (14.6) | 169 (14.9) | 146 (12.7) | 133 (11.6) |  |
| **Thoracic surgery** | 311 (6.8) | 65 (5.7) | 74 (6.5) | 91 (7.9) | 81 (7.1) |  |
| **Gynecology** | 186 (4.1) | 46 (4.0) | 46 (4.0) | 49 (4.3) | 45 (3.9) |  |
| **E.N.T** | 290 (6.4) | 78 (6.9) | 80 (7.0) | 72 (6.3) | 60 (5.3) |  |
| **Vascular surgery** | 229 (5.0) | 72 (6.3) | 53 (4.7) | 48 (4.2) | 56 (4.9) |  |
| **Others** | 1481 (32.4) | 337 (29.6) | 350 (30.8) | 381 (33.2) | 413 (36.2) |  |
| **Anesthesia type, n (%)** |  |  |  |  |  | 0.042 |
| **General anesthesia** | 4038 (88.4) | 1009 (88.6%) | 1017 (89.5%) | 1019 (88.8%) | 993 (87.0%) |  |
| **Basal anesthesia** | 150 (3.3) | 41 (3.6%) | 35 (3.1%) | 21 (1.8%) | 53 (4.6%) |  |
| **General anesthesia combined with other anesthesia** | 288 (6.3) | 71 (6.2%) | 59 (5.2%) | 83 (7.2%) | 75 (6.6%) |  |
| **Epidural anesthesia** | 45 (1.0) | 11 (1.0%) | 13 (1.1%) | 12 (1.1%) | 9 (0.8%) |  |
| **Nerve blocks** | 45 (1.0) | 7 (0.6%) | 13 (1.1%) | 13 (1.1%) | 12 (1.0%) |  |
| **GSP, μmol/L** | 200 [178-226] | 196 [168-210] | 200 [173-217] | 200 [184-228] | 205 [198-250] | <0.001 |
| **Age, years** | 70.0 [67.0-74.0] | 71.0 [68.0-75.0] | 71.0 [67.0-75.0] | 70.0 [67.0-74.0] | 70.0 [67.0-74.0] | 0.001 |
| **ALT, U/L** | 14.9 [11.0-22.2] | 14.1 [10.3-19.9] | 14.2 [10.7-20.4] | 14.4 [11.1-21.4] | 17.9 [12.3-26.6] | <0.001 |
| **AST, U/L** | 16.1 [13.3-20.6] | 16.2 [13.6-20.2] | 15.9 [13.2-19.6] | 15.9 [13.0-20.2] | 16.8 [13.5-22.8] | <0.001 |
| **BMI, kg/m^2^** | 24.8 [22.7-27.1] | 23.8 [21.6-25.9] | 24.8 [22.7-27.0] | 25.2 [23.2-27.6] | 25.4 [23.2-27.5] | <0.001 |
| **Hb, g/L** | 130 [119-141] | 128 [117-138] | 130 [119-142] | 131 [119-142] | 130 [120-141] | <0.001 |
| **WBC count, *10^9^/L** | 6.04 [5.07-7.28] | 5.65 [4.78-6.86] | 5.99 [5.07-7.16] | 6.19 [5.23-7.36] | 6.34 [5.29-7.68] | <0.001 |
| **Total bilirubin, μmol/L** | 10.5 [8.00-14.0] | 10.8 [8.40-13.9] | 10.8 [8.30-14.1] | 10.4 [7.90-13.7] | 9.95 [7.40-14.3] | 0.006 |
| **PT, s** | 13.2 [12.7-13.8] | 13.4 [12.9-14.0] | 13.3 [12.8-13.8] | 13.1 [12.6-13.7] | 13.0 [12.5-13.5] | <0.001 |
| **Duration of anesthesia, min** | 180 [115-245] | 180 [110-244] | 182 [118-240] | 177 [115-250] | 182 [120-250] | 0.551 |
| **Blood loss, ml** | 80 [20-200] | 50 [20-200] | 100 [20-200] | 50 [20-200] | 100 [20-200] | 0.704 |
| **Urine, ml** | 200 [50-500] | 200 [50-500] | 200 [50-500] | 200 [50-500] | 200 [50-500] | 0.978 |
| **Crystalloid, ml** | 1350 [1000-2100] | 1450 [1000-2100] | 1400 [1000-2100] | 1300 [1000-2100] | 1325 [1000-2100] | 0.905 |
| **Colloid, ml** | 500 [0-500] | 50 [20-200] | 100 [20-200] | 50 [20-200] | 100 [20-200] | 0.704 |
| **Cre, μmol/L** | 71.2 [59.8-83.7] | 72.3 [61.5-83.5] | 72.1 [60.7-85.2] | 70.4 [59.7-81.7] | 69.8 [57.6-84.3] | 0.001 |
| **Total cholesterol, mmol/L** | 4.22 [3.60-4.94] | 3.89 [3.29-4.44] | 4.14 [3.51-4.79] | 4.32 [3.73-5.05] | 4.64 [3.98-5.42] | <0.001 |
| **LDL, mmol/L** | 2.64 [2.16-3.23] | 2.42 [1.90-2.86] | 2.64 [2.16-3.18] | 2.71 [2.29-3.40] | 2.71 [2.30-3.43] | <0.001 |
| **HDL, mmol/L** | 1.10 [0.93-1.29] | 1.22 [1.04-1.46] | 1.10 [0.97-1.30] | 1.10 [0.92-1.25] | 1.01 [0.83-1.14] | <0.001 |
| **Glucose, mmol/L** | 5.90 [4.96-7.34] | 4.82 [4.33-5.45] | 5.53 [4.90-6.39] | 6.47 [5.49-7.55] | 7.80 [6.38-9.92] | <0.001 |
| **Triglyceride, mmol/L** | 1.27 [0.94-1.76] | 0.79 [0.66-0.93] | 1.15 [0.99-1.32] | 1.48 [1.26-1.73] | 2.21 [1.77-2.84] | <0.001 |
| **Duration of MAP<60 mmHg, min** | 5.0 [0.0-10.0] | 5.0 [0.0-15.0] | 5.0 [0.0-10.0] | 5.0 [0.0-10.0] | 5.0 [0.0-10.0] | 0.252 |
| **Platelet count, *10^9^/L** | 207 [170-252] | 205 [168-249] | 209 [169-252] | 209 [170-251] | 207 [172-255] | 0.436 |

POD, postoperative delirium; COPD, chronic obstructive pulmonary disease; CKD, chronic kidney disease; ASA, [American Society of Anesthesiologists;](https://www.medsci.cn/guideline/search?keyword=美国麻醉医师协会(ASA,American Society of Anesthesiologists)) E.N.T., Otolaryngology head, and neck surgery; GSP, glycated **s**erum protein; ALT, alanine aminotransferase; AST, aspartate aminotransferase; BMI, body mass index; Hb, hemoglobin; WBC, white blood cell; PT, prothrombin time; TyG, triglyceride-glucose; Cre, Creatinine; LDL, low density lipoprotein; HDL, high density lipoprotein; MAP, mean artery pressure.
