# Supplementary material for: Triglyceride-glucose index predicts postoperative delirium in elderly patients with type 2 diabetes mellitus: a retrospective cohort study
Source: Lipids Health Dis. 2024 Apr 15;23:107. doi: 10.1186/s12944-024-02084-2 (PMC11017528; doi:10.1186/s12944-024-02084-2)
Supplement: Supplementary file 3 — Supplementary Material 3 [file 12944_2024_2084_MOESM3_ESM.doc]

**Supplementary table 3. Univariate logistic regression analysis of variables for POD**

| **Characteristics** | **Estimate** | ***P* value** | **OR(95%CI)** |
| --- | --- | --- | --- |
| **TyG as a continuous variable** | 0.322 | 0.008 | 1.379 (1.084 -1.749 ) |
| **TyG > 8.678** | 0.512 | 0.002 | 1.668 (1.210 -2.324 ) |
| **TyG index quartiles (TyG ≤ 8.338 as reference)** | | | |
| **8.338 < TyG ≤ 8.736** | 0.245 | 0.322 | 1.278 (0.788 -2.092 ) |
| **8.736 < TyG ≤ 9.171** | 0.456 | 0.055 | 1.578 (0.996 -2.538 ) |
| **TyG > 9.171** | 0.547 | 0.019 | 1.728 (1.100 -2.762 ) |
| **Gender (female)** | -0.175 | 0.274 | 0.840 (0.612 -1.147 ) |
| **Smoking** | -0.024 | 0.895 | 0.976 (0.672 -1.389 ) |
| **Alcohol** | -0.207 | 0.307 | 0.813 (0.538 -1.192 ) |
| **Hypertension** | 0.098 | 0.568 | 1.103 (0.792 -1.558 ) |
| **Cardiac disease** | 0.159 | 0.378 | 1.172 (0.830 -1.686 ) |
| **COPD** | 0.536 | 0.110 | 1.710 (0.830 -3.143 ) |
| **Cerebrovascular disease** | 0.360 | 0.082 | 1.433 (0.939 -2.117 ) |
| **CKD** | 1.438 | 0.000 | 4.212 (2.202 -7.452 ) |
| **Depression and anxiety** | 1.345 | 0.031 | 3.838 (0.900 -11.274 ) |
| **Emergency surgery** | 0.745 | 0.028 | 2.106 (1.019 -3.889 ) |
| **Surgery types (Hepatopancreatobiliary and gastrointestinal surgery as reference)** | | | |
| **Urinary surgery** | -0.474 | 0.071 | 0.622 (0.362 -1.020 ) |
| **Thoracic surgery** | -0.958 | 0.026 | 0.383 (0.148 -0.820 ) |
| **Gynecology** | -0.431 | 0.319 | 0.650 (0.249 -1.399 ) |
| **E.N.T** | -1.300 | 0.012 | 0.273 (0.083 -0.664 ) |
| **Vascular surgery** | -1.762 | 0.014 | 0.172 (0.028 -0.551 ) |
| **Others** | -0.230 | 0.204 | 0.795 (0.556-1.132) |
| **Anesthesia types (General anesthesia as reference)** | | | |
| **Basal anesthesia** | -0.342 | 0.505 | 0.710 (0.216 -1.710 ) |
| **General anesthesia combined with other anesthesia** | 0.120 | 0.696 | 1.127 (0.586 -1.970 ) |
| **Epidural anesthesia** | -14.311 | 0.981 | 0.000 (0.000 -58080.750 ) |
| **Nerve blocks** | -14.311 | 0.981 | 0.000 (0.000 -58080.750 ) |
| **GSP** | 0.000 | 0.768 | 1.000 (0.996 -1.003 ) |
| **Age** | 0.056 | 0.000 | 1.058 (1.029 -1.086 ) |
| **ALT** | -0.003 | 0.343 | 0.997 (0.990 -1.002 ) |
| **AST** | 0.000 | 0.907 | 1.000 (0.993 -1.004 ) |
| **BMI** | -0.021 | 0.347 | 0.980 (0.939 -1.023 ) |
| **Hb** | -0.023 | <0.001 | 0.977 (0.970 -0.984 ) |
| **WBC count** | 0.099 | <0.001 | 1.104 (1.052 -1.163 ) |
| **Total bilirubin** | 0.000 | 0.970 | 1.000 (0.995 -1.004 ) |
| **PT** | 0.061 | 0.303 | 1.063 (0.937 -1.178 ) |
| **Duration of anesthesia** | 0.004 | <0.001 | 1.004 (1.003 -1.006 ) |
| **Blood loss** | 0.001 | <0.001 | 1.001 (1.001 -1.001 ) |
| **Urine** | 0.001 | <0.001 | 1.001 (1.000 -1.001 ) |
| **Crystalloid** | 0.000 | <0.001 | 1.000 (1.000 -1.001 ) |
| **Colloid** | 0.001 | <0.001 | 1.001 (1.001 -1.001 ) |
| **Cre** | 0.001 | 0.422 | 1.001 (0.998 -1.004 ) |
| **Total cholesterol** | -0.092 | 0.208 | 0.912 (0.787 -1.046 ) |
| **LDL** | -0.082 | 0.379 | 0.921 (0.765 -1.103 ) |
| **HDL** | -1.178 | 0.000 | 0.308 (0.182 -0.518 ) |
| **Glucose** | 0.098 | 0.000 | 1.103 (1.046 -1.158 ) |
| **Triglyceride** | 0.023 | 0.793 | 1.023 (0.853 -1.201 ) |
| **Duration of MAP<60 mmHg** | 0.012 | <0.001 | 1.012 (1.006 -1.018 ) |
| **RBC count** | -0.003 | 0.018 | 0.997 (0.995-0.999) |

POD, postoperative delirium; COPD, chronic obstructive pulmonary disease; CKD, chronic kidney disease; ASA, [American Society of Anesthesiologists;](https://www.medsci.cn/guideline/search?keyword=美国麻醉医师协会(ASA,American Society of Anesthesiologists)) E.N.T., Otolaryngology head, and neck surgery; GSP, glycated serum protein; ALT, alanine aminotransferase; AST, aspartate aminotransferase; BMI, body mass index; Hb, hemoglobin; WBC, white blood cell; PT, prothrombin time; TyG, triglyceride-glucose; Cre,Creatinine; LDL, low density lipoprotein; HDL, high density lipoprotein; MAP, mean artery pressure.
