# Supplementary material for: Triglyceride-glucose index predicts postoperative delirium in elderly patients with type 2 diabetes mellitus: a retrospective cohort study
Source: Lipids Health Dis. 2024 Apr 15;23:107. doi: 10.1186/s12944-024-02084-2 (PMC11017528; doi:10.1186/s12944-024-02084-2)
Supplement: Supplementary file 4 — Supplementary Material 4 [file 12944_2024_2084_MOESM4_ESM.doc]

**Supplementary table 4. Association between POD and TyG as a binary variable in different models**

| **Variables** | **Model 1** | | **Model 2** | | **Model 3** | |
| --- | --- | --- | --- | --- | --- | --- |
|  | **OR(95%CI)** | ***P* value** | **OR(95%CI)** | ***P* value** | **OR(95%CI)** | ***P* value** |
| **TyG > 8.678** | 1.590 (1.133 -2.252 ) | 0.008 | 1.661 (1.199 -2.325 ) | 0.003 | 1.603 (1.137 -2.283 ) | 0.008 |
| **CKD** | 2.745 (1.396 -5.005 ) | 0.002 |  |  | 3.280 (1.645 -6.102 ) | <0.001 |
| **Depression and anxiety** | 3.744 (0.859 -11.416 ) | 0.038 |  |  | 3.254 (0.713 -10.646 ) | 0.078 |
| **Age** | 1.045 (1.016 -1.074 ) | 0.002 |  |  | 1.062 (1.031 -1.094 ) | <0.001 |
| **Hb** | 0.981 (0.974 -0.989 ) | 0.000 |  |  | 0.983 (0.975 -0.991 ) | <0.001 |
| **WBC count** | 1072 (1.023 -1.125 ) | 0.003 |  |  | 1.059 (1.009 -1.115 ) | 0.021 |
| **HDL** | 0.504 (0.291 -0.868 ) | 0.014 |  |  | 0.511 (0.291 -0.889 ) | 0.018 |
| **Platelet count** | 0.997 (0.995 -0.999 ) | 0.017 |  |  | 0.997 (0.994 -0.999 ) | 0.014 |
| **Emergency surgery** |  |  | 2.831 (1.317 -5.499 ) | 0.004 | 2.100 (0.941 -4.236 ) | 0.051 |
| **Surgery types (Hepatopancreatobiliary and gastrointestinal surgery as reference)** | | | | | | |
| **Urinary surgery** |  |  | 0.827 (0.472 -1.386 ) | 0.487 | 0.978 (0.551 -1.664 ) | 0.935 |
| **Thoracic surgery** |  |  | 0.441 (0.167 -0.969 ) | 0.063 | 0.683 (0.255 -1.532 ) | 0.397 |
| **Gynecology** |  |  | 0.879 (0.330 -1.953 ) | 0.772 | 1.107 (0.410 -2.501 ) | 0.823 |
| **E.N.T** |  |  | 0.419 (0.124 -1.064 ) | 0.104 | 0.657 (0.192 -1.707 ) | 0.440 |
| **Vascular surgery** |  |  | 0.253 (0.041 -0.845 ) | 0.061 | 0.185 (0.028 -0.674 ) | 0.030 |
| **Others** |  |  | 0.914 (0.595 -1.401 ) | 0.680 | 1.155 (0.736 -1.812 ) | 0.529 |
| **Duration of anesthesia** |  |  | 1.003 (1.000 -1.005 ) | 0.027 | 1.003 (1.000 -1.005 ) | 0.038 |
| **Blood loss** |  |  | 1.000 (1.000 -1.001 ) | 0.337 | 1.000 (1.000 -1.001 ) | 0.575 |
| **Urine** |  |  | 1.000 (1.000 -1.001 ) | 0.173 | 1.000 (1.000 -1.001 ) | 0.171 |
| **Crystalloid** |  |  | 1.000 (1.000 -1.00 ) | 0.600 | 1.000 (1.000 -1.000 ) | 0.960 |
| **Colloid** |  |  | 1.000 (1.000 -1.001 ) | 0.436 | 1.000 (1.000 -1.001 ) | 0.230 |
| **Duration of MAP<60 mmHg** |  |  | 1.006 (0.999 -1.012 ) | 0.089 | 1.004 (0.996 -1.010 ) | 0.325 |

TyG, triglyceride-glucose; POD, postoperative delirium; CKD, chronic kidney disease; E.N.T., Otolaryngology head, and neck surgery; GSP, glycated serum protein; Hb, hemoglobin; WBC, white blood cell; HDL, high density lipoprotein; MAP, mean artery pressure.
