# Supplementary material for: Triglyceride-glucose index predicts postoperative delirium in elderly patients with type 2 diabetes mellitus: a retrospective cohort study
Source: Lipids Health Dis. 2024 Apr 15;23:107. doi: 10.1186/s12944-024-02084-2 (PMC11017528; doi:10.1186/s12944-024-02084-2)
Supplement: Supplementary file 5 — Supplementary Material 5 [file 12944_2024_2084_MOESM5_ESM.doc]

**Supplementary table 5. Association between POD and TyG as a continuous variable in different models**

| **Variables** | **Model 1** | | **Model 2** | | **Model 3** | |
| --- | --- | --- | --- | --- | --- | --- |
|  | **OR(95%CI)** | ***P* value** | **OR(95%CI)** | ***P* value** | **OR(95%CI)** | ***P* value** |
| **TyG** | 1.268 (0.977 -1.640 ) | 0.072 | 1.335 (1.047 -1.698 ) | 0.019 | 1.255 (0.962 -1.631 ) | 0.092 |
| **CKD** | 2.713 (1.379 -4.951 ) | 0.002 |  |  | 3.294 (0.721 -10.774 ) | 0.075 |
| **Depression and anxiety** | 3.766 (0.864 -11.480 ) | 0.038 |  |  | 3.254 (0.713 -10.646 ) | 0.078 |
| **Age** | 1.045 (1.016 -1.074 ) | 0.002 |  |  | 1.062 (1.030 -1.094 ) | <0.001 |
| **Hb** | 0.982 (0.974 -0.989 ) | 0.000 |  |  | 0.983 (0.976 -0.992 ) | <0.001 |
| **WBC count** | 1.071 (1.022 -1.124 ) | 0.003 |  |  | 1.058 (1.009 -1.115 ) | 0.023 |
| **HDL** | 0.500 (0.285 -0.872 ) | 0.015 |  |  | 0.500 (0.282 -0.881 ) | 0.017 |
| **Platelet count** | 0.997 (0.995 -0.999 ) | 0.017 |  |  | 0.997 (0.994 -0.999 ) | 0.015 |
| **Emergency surgery** |  |  | 2.859 (1.333 -5.545 ) | 0.004 | 2.094 (0.938 -4.224 ) | 0.052 |
| **Surgery types (Hepatopancreatobiliary and gastrointestinal surgery as reference)** | | | | | | |
| **Urinary surgery** |  |  | 0.823 (0.470 -1.379 ) | 0.476 | 0.973 (0.548 -1.654 ) | 0.921 |
| **Thoracic surgery** |  |  | 0.454 (0.172 -0.995 ) | 0.073 | 0.701 (0.262 -1.571 ) | 0.429 |
| **Gynecology** |  |  | 0.885 (0.332 -1.967 ) | 0.783 | 1.128 (0.419 -2.547 ) | 0.791 |
| **E.N.T** |  |  | 0.411 (0.040 -0.835 ) | 0.096 | 0.649 (0.190 -1.684 ) | 0.426 |
| **Vascular surgery** |  |  | 0.250 (0.122 -1.044 ) | 0.059 | 0.187 (0.028 -0.680 ) | 0.031 |
| **Others** |  |  | 0.918 (0.598 -1.408 ) | 0.696 | 1.172 (0.747 -1.839 ) | 0.488 |
| **Duration of anesthesia** |  |  | 1.003 (1.000 -1.005 ) | 0.028 | 1.003 (1.000 -1.005 ) | 0.036 |
| **Blood loss** |  |  | 1.000 (1.000 -1.001 ) | 0.366 | 1.000 (1.000 -1.001 ) | 0.636 |
| **Urine** |  |  | 1.000 (1.000 -1.001 ) | 0.186 | 1.000 (1.000 -1.001 ) | 0.195 |
| **Crystalloid** |  |  | 1.000 (1.000 -1.000 ) | 0.611 | 1.000 (1.000 -1.000 ) | 0.952 |
| **Colloid** |  |  | 1.000 (1.000 -1.001 ) | 0.444 | 1.000 (1.000 -1.001 ) | 0.221 |
| **Duration of MAP<60 mmHg** |  |  | 1.006 (0.999 -1.012 ) | 0.089 | 1.004 (0.996 -1.010 ) | 0.319 |

TyG, triglyceride-glucose; POD, postoperative delirium; CKD, chronic kidney disease; E.N.T., Otolaryngology head, and neck surgery; GSP, glycated serum protein; Hb, hemoglobin; WBC, white blood cell; HDL, high density lipoprotein; MAP, mean artery pressure.
