# Supplementary material for: Triglyceride-glucose index predicts postoperative delirium in elderly patients with type 2 diabetes mellitus: a retrospective cohort study
Source: Lipids Health Dis. 2024 Apr 15;23:107. doi: 10.1186/s12944-024-02084-2 (PMC11017528; doi:10.1186/s12944-024-02084-2)
Supplement: Supplementary file 6 — Supplementary Material 6 [file 12944_2024_2084_MOESM6_ESM.doc]

**Supplementary table 6. Association between POD and TyG as a quartiles variable in different models**

| **Variables** | **Model 1** | | **Model 2** | | **Model 3** | |
| --- | --- | --- | --- | --- | --- | --- |
|  | **OR(95%CI)** | ***P* value** | **OR(95%CI)** | ***P* value** | **OR(95%CI)** | ***P* value** |
| **TyG ≤ 8.338 as reference** | | | | | | |
| **8.338 < TyG ≤ 8.736** | 1.216 (0.740 -2.015 ) | 0.442 | 1.270 (0.780 -2.087 ) | 0.339 | 1.194 (0.723 -1.988 ) | 0.489 |
| **8.736 < TyG ≤ 9.171** | 1.549 (0.963 -2.527 ) | 0.074 | 1.592 (1.000 -2.570 ) | 0.053 | 1.519 (0.940 -2.492 ) | 0.092 |
| **TyG > 9.171** | 1.518 (0.936 -2.498 ) | 0.095 | 1.666 (1.055 -2.677 ) | 0.031 | 1.489 (0.913 -2.464 ) | 0.115 |
| **CKD** | 2.764 (1.407 -2.086 ) | 0.037 |  |  | 3.269 (1.640 -6.080 ) | 0.000 |
| **Depression and anxiety** | 3.869 (0.887 -11.808 ) | 0.034 |  |  | 3.353 (0.734 -10.982 ) | 0.071 |
| **Age** | 1.045 (1.016 -1.074 ) | 0.002 |  |  | 3.353 (0.734 -10.982 ) | 0.071 |
| **Hb** | 0.982 (0.974 -0.989 ) | 0.000 |  |  | 0.983 (0.976 -0.991 ) | 0.000 |
| **WBC count** | 1.072 (1.023 -1.125 ) | 0.003 |  |  | 1.058 (1.009 -1.115 ) | 0.024 |
| **HDL** | 0.488 (0.279 -0.848 ) | 0.011 |  |  | 0.490 (0.277 -0.860 ) | 0.014 |
| **Platelet count** | 0.997 (0.995 -0.999 ) | 0.017 |  |  | 0.997 (0.994 -0.999 ) | 0.015 |
| **Emergency surgery** |  |  | 2.892 (1.347 -5.612 ) | 0.003 | 2.126 (0.953 -4.287 ) | 0.047 |
| **Surgery types (Hepatopancreatobiliary and gastrointestinal surgery as reference)** | | | | | | |
| **Urinary surgery** |  |  | 0.827 (0.472 -1.385 ) | 0.486 | 0.977 (0.551 -1.662 ) | 0.934 |
| **Thoracic surgery** |  |  | 0.449 (0.170 -0.986 ) | 0.069 | 0.696 (0.260 -1.560 ) | 0.420 |
| **Gynecology** |  |  | 0.890 (0.334 -1.978 ) | 0.794 | 1.127 (0.418 -2.545 ) | 0.792 |
| **E.N.T** |  |  | 0.415 (0.123 -1.055 ) | 0.100 | 0.652 (0.190 -1.692 ) | 0.431 |
| **Vascular surgery** |  |  | 0.256 (0.041 -0.854 ) | 0.063 | 0.193 (0.029 -0.697 ) | 0.033 |
| **Others** |  |  | 0.921 (0.600 -1.412 ) | 0.706 | 1.173 (0.748 -1.840 ) | 0.487 |
| **Duration of anesthesia** |  |  | 1.003 (1.000 -1.005 ) | 0.027 | 1.003 (1.000 -1.005 ) | 0.035 |
| **Blood loss** |  |  | 1.000 (1.000 -1.001 ) | 0.371 | 1.000 (1.000 -1.001 ) | 0.626 |
| **Urine** |  |  | 1.000 (1.000 -1.001 ) | 0.192 | 1.000 (1.000 -1.001 ) | 0.192 |
| **Crystalloid** |  |  | 1.000 (1.000 -1.000 ) | 0.620 | 1.000 (1.000 -1.000 ) | 0.960 |
| **Colloid** |  |  | 1.000 (1.000 -1.001 ) | 0.422 | 1.000 (1.000 -1.001 ) | 0.216 |
| **Duration of MAP<60 mmHg** |  |  | 1.006 (0.999 -1.012 ) | 0.085 | 1.004 (0.996 -1.010 ) | 0.317 |

TyG, triglyceride-glucose; POD, postoperative delirium; CKD, chronic kidney disease; E.N.T., Otolaryngology head, and neck surgery; GSP, glycated serum protein; Hb, hemoglobin; WBC, white blood cell; HDL, high density lipoprotein; MAP, mean artery pressure.
