# Supplementary material for: Triglyceride-glucose index predicts postoperative delirium in elderly patients with type 2 diabetes mellitus: a retrospective cohort study
Source: Lipids Health Dis. 2024 Apr 15;23:107. doi: 10.1186/s12944-024-02084-2 (PMC11017528; doi:10.1186/s12944-024-02084-2)
Supplement: Supplementary file 7 — Supplementary Material 7 [file 12944_2024_2084_MOESM7_ESM.doc]

**Supplementary table 7. Clinical characteristics of participants by gender**

| **Characteristics** | **Male** | **Female** | ***P* value** |
| --- | --- | --- | --- |
| **Number, n (%)** | 2423 (53.1) | 2143 (46.9) |  |
| **TyG** | 8.65 [8.26-9.09] | 8.84 [8.44-9.25] | <0.001 |
| **POD, n (%)** |  |  | 0.31 |
| no | 2328 (96.1) | 2072 (96.7) |  |
| yes | 95 (3.9) | 71 (3.3) |  |
| **Smoking, n (%)** |  |  | <0.001 |
| no | 1380 (57.0) | 2066 (96.4) |  |
| yes | 1043 (43.0) | 77 (3.6) |  |
| **Alcohol, n (%)** |  |  | <0.001 |
| no | 1476 (60.9) | 2090 (97.5) |  |
| yes | 947 (39.1) | 53 (2.5) |  |
| **Hypertension, n (%)** |  |  | <0.001 |
| no | 856 (35.3) | 612 (28.6) |  |
| yes | 1567 (64.7) | 1531 (71.4) |  |
| **Cardiac disease, n (%)** |  |  | <0.001 |
| no | 765 (31.6) | 557 (26.0) |  |
| yes | 1658 (68.4) | 1586 (74.0) |  |
| **COPD, n (%)** |  |  | <0.001 |
| no | 2309 (95.3) | 2088 (97.4) |  |
| yes | 114 (4.7) | 55 (2.6) |  |
| **Cerebrovascular disease, n (%)** |  |  | 0.117 |
| no | 2077 (85.7) | 1872 (87.4) |  |
| yes | 346 (14.3) | 271 (12.6) |  |
| **CKD, n (%)** |  |  | 0.93 |
| no | 2369 (97.8) | 2097 (97.9) |  |
| yes | 54 (2.2) | 46 (2.1) |  |
| **Depression and anxiety, n (%)** |  |  | 0.185 |
| no | 2414 (99.6) | 2128 (99.3) |  |
| yes | 9 (0.4) | 15 (0.7) |  |
| **ASA grade, n (%)** |  |  | 0.793 |
| Ⅰ | 21 (0.9) | 19 (0.9) |  |
| Ⅱ | 1778 (73.4) | 1551 (72.4) |  |
| Ⅲ | 606 (25.0) | 560 (26.1) |  |
| Ⅳ | 18 (0.7) | 13 (0.6) |  |
| **Emergency surgery, n (%)** |  |  | 0.758 |
| no | 2351 (97.0) | 2075 (96.8) |  |
| yes | 72 (3.0) | 68 (3.2) |  |
| **Surgical type, n (%)** |  |  | <0.001 |
| **Hepatopancreatobiliary and**  **gastrointestinal surgery** | 915 (37.8) | 540 (25.2) |  |
| **Urinary surgery** | 433 (17.9) | 181 (8.4) |  |
| **Thoracic surgery** | 190 (7.8) | 121 (5.6) |  |
| **Gynecology** | 0 (0.0) | 186 (8.7) |  |
| **E.N.T** | 166 (6.9) | 124 (5.8) |  |
| **Vascular surgery** | 150 (6.2) | 79 (3.7) |  |
| **Others** | 569 (23.4) | 912 (42.6) |  |
| **Anesthesia type, n (%)** |  |  | <0.001 |
| **General anesthesia** | 2204 (91.0) | 1834 (85.6) |  |
| **Basal anesthesia** | 85 (3.5) | 65 (3.0) |  |
| **General anesthesia combined with other anesthesia** | 96 (4.0) | 192 (9.0) |  |
| **Epidural anesthesia** | 21 (0.8) | 24 (1.1) |  |
| **Nerve blocks** | 17 (0.7) | 28 (1.3) |  |
| **GSP, μmol/L** | 200 [179-229] | 200 [177-222] | 0.001 |
| **Age, years** | 70.0 [67.0-74.0] | 70.0 [67.0-75.0] | 0.26 |
| **ALT, U/L** | 15.7 [11.5-23.4] | 14.1 [10.4-20.6] | <0.001 |
| **AST, U/L** | 16.0 [13.4-20.6] | 16.2 [13.3-20.5] | 0.782 |
| **BMI, kg/m^2^** | 24.6 [22.5-26.8] | 25.0 [22.8-27.5] | <0.001 |
| **Hb, g/L** | 136 [123-146] | 125 [116-133] | <0.001 |
| **WBC count, *10^9^/L** | 6.13 [5.13-7.39] | 5.94 [5.00-7.18] | 0.001 |
| **Total bilirubin, μmol/L** | 11.30 [8.60-15.2] | 9.70 [7.55-12.6] | <0.001 |
| **PT, s** | 13.3 [12.8-13.9] | 13.1 [12.6-13.6] | <0.001 |
| **Duration of anesthesia, min** | 190 [125-258] | 170 [108-232] | <0.001 |
| **Blood loss, ml** | 80 [20-200] | 80 [20-200] | 0.532 |
| **Urine, ml** | 200 [55-500] | 200 [50-500] | 0.72 |
| **Crystalloid, ml** | 1600 [1100-2100] | 1200 [1000-1700] | <0.001 |
| **Colloid, ml** | 500 [0.00-500] | 500 [0.00-500] | <0.001 |
| **Cre, μmol/L** | 78.7 [69.8-89.8] | 61.2 [54.0-71.5] | <0.001 |
| **Total cholesterol** | 4.01 [3.44-4.64] | 4.51 [3.85-5.27] | <0.001 |
| **LDL, mmol/L** | 2.59 [2.06-3.03] | 2.73 [2.27-3.41] | <0.001 |
| **HDL, mmol/L** | 1.07 [0.89-1.23] | 1.13 [0.98-1.37] | <0.001 |
| **Glucose, mmol/L** | 5.84 [4.94-7.33] | 5.96 [4.98-7.36] | 0.222 |
| **Triglyceride, mmol/L** | 1.18 [0.88-1.59] | 1.40 [1.04-1.90] | <0.001 |
| **Duration of MAP<60 mmHg, min** | 5.0 [0.0-10.0] | 5.0 [0.0-10.0] | 0.447 |
| **Platelet count, *10^9^/L** | 207 [169-251] | 208 [170-253] | 0.272 |

POD, postoperative delirium; COPD, chronic obstructive pulmonary disease; CKD, chronic kidney disease; ASA, [American Society of Anesthesiologists;](https://www.medsci.cn/guideline/search?keyword=美国麻醉医师协会(ASA,American Society of Anesthesiologists)) E.N.T., Otolaryngology head, and neck surgery; GSP, glycated serum protein; ALT, alanine aminotransferase; AST, aspartate aminotransferase; BMI, body mass index; Hb, hemoglobin; WBC, white blood cell; PT, prothrombin time; TyG, triglyceride-glucose; Cre,Creatinine; LDL, low density lipoprotein; HDL, high density lipoprotein; MAP, mean artery pressure.
