# Supplementary material for: Triglyceride-glucose index predicts postoperative delirium in elderly patients with type 2 diabetes mellitus: a retrospective cohort study
Source: Lipids Health Dis. 2024 Apr 15;23:107. doi: 10.1186/s12944-024-02084-2 (PMC11017528; doi:10.1186/s12944-024-02084-2)
Supplement: Supplementary file 8 — Supplementary Material 8 [file 12944_2024_2084_MOESM8_ESM.doc]

**Supplementary table 8. Sensitivity analysis of association between TyG index and POD with logistic regression models**

| **Model** | **OR^a^** | **95%CI** | ***P* value** |
| --- | --- | --- | --- |
| **Unadjusted model** | 1.593 | 1.146 -2.238 | 0.006 |
| **Model 1 (adjusted for preoperative variables)** | 1.493 | 1.054 -2.133 | 0.025 |
| **Model 2 (adjusted for intraoperative variables)** | 1.648 | 1.763 -2.331 | 0.004 |
| **Model 3 (adjusted for all the variables)** | 1.582 | 1.108 -2.279 | 0.012 |

OR^a^ is the odd ratio of TyG > 8.678; Preoperative variables include CKD, depression and anxiety, age, Hb, WBC, HDL, platelet count; Intraoperative variables include emergency surgery, surgery types, anesthesia time, blood loss, urine, Duration of MAP<60 mmHg, Crystalloid, Colloid.
